# Researches into the Physical Causes of Metallic Tinkling, or Amphoric Rhonchus

**Published:** 1842-08

**Authors:** 

**Affiliations:** House Surgeon (interne) of the Hospitals


*Researches into the Physical Causes of Metallic Tinkling, or Amphoric Rhonchus.* By. M. de Castelnau, House Surgeon (interne) of the Hospitals.—The author commences this paper by reviewing the different theories which have been suggested to explain this phenomenon, and pronounces them all more or less unsatisfactory. The hypothesis most usually adopted, and which attributes the sound to the bursting of an air-bubble on the surface of the fluid effused into the pleural cavity, is regarded by him as equally defective with the others. Experiments which he details have led him to the conclusion, that the formation of bubbles of air at the surface of an effusion is almost impossible, even in those cases where the perforation of the pleura is below the level of the fluid, while its occurrence is altogether out of the question when there exist perforations of the pleura above the level of the effusion.

The occurrence of pulmonary fistula, however, above the level of an effusion is by no means unusual; it is even stated by M. Raciborski to be the case in by far the greater number of instances. Laennec, too, had observed that, after the operation for empyema, in which the puncture is made above the level of the fluid, metallic tinkling is frequently heard; while if the wound had been made too large, the respiration acquires an amphoric sound. This phenomenon can be ex-

6*

plained only by supposing metallic tinkling to be a variety of the amphoric sound; and M. C's experiments on the dead subject have convinced him that such is really the case. He further deduces from them the following conclusions:

1. That the physical conditions essential to the production of metallic tinkling are: *a*, The existence of a tolerably large cavity, containing air, either with or without fluid; *b*, The communication of the external air with this cavity; *c*, The production of sonorous vibrations in the channels by which this communication is established.

2. The causes which gave rise to these vibrations are identical with those which produce moist sounds in general.

3. Metallic tinkling may be called an amphoric rhonchus, with as much propriety as the term amphoric may be applied to the respiration, voice, or cough.

4. Those cases, if indeed any such exist, in which metallic tinkling occurs independent of the above-mentioned conditions, are exceptions to the rule, as are also the theories advanced in explanation of them.

In confirmation of these views, a case is related in which metallic tinkling was heard, and the respiration, voice and cough had an amphoric sound in a phthisical patient. After death the left lung was found to be occupied by two very large cavities, which destroyed the greater part of its substance. A septum, only three or four lines thick, separated the two cavities from each other. The superior was empty; the other contained about four ounces of broken down tuberculous matter, in a semi-fluid rather than a liquid state, and the openings of the bronchi into the cavity were all, with the exception of two, situated above the level of the softened tubercle. Similar phenomenon were observed in the case of a man in whom fracture of the ribs and clavicle was followed by subcutaneous emphysema and pneumothorax. The metallic tinkling and amphoric respiration disappeared gradually as the man advanced towards convalescence; and there was no reason, at any period of his illness, to suppose the existence of fluid in cavity of the pleura. He likewise alludes to a third case, in which a wound in the chest with a knife, though unaccompanied with subcutaneous emphysema, or even of hæmoptysis, was followed by metallic tinkling and amphoric sounds.

In a second paper on the same subject, two other cases are adduced in confirmation of the author's views.—*Archives Générales de Médecine. Oct. et Nov.* 1841.

